# Global burden and socioeconomic impact of knee osteoarthritis: a comprehensive analysis

**DOI:** 10.3389/fmed.2024.1323091

**Published:** 2024-05-16

**Authors:** Erliang Li, Jianshi Tan, Ke Xu, Ying Pan, Peng Xu

**Affiliations:** ^1^Department of Joint Surgery, Honghui Hospital, Xi'an Jiaotong University, Xi'an, China; ^2^Department of Orthopedics, The First Affiliated Hospital of Xi'an Jiaotong University, Xi'an, China; ^3^Department of Orthopedics, The Second Affiliated Hospital of Guangxi Medical University, Nanning, China

**Keywords:** Global Burden of Disease, knee osteoarthritis, prevalence, incidence, disability-adjusted life years (DALYs)

## Abstract

**Objective:**

To report the trend changes of the prevalence, incidence, and disability-adjusted life years (DALYs) of knee osteoarthritis (KOA) according to age, sex, sociodemographic index (SDI), and income.

**Methods:**

This analysis used estimates from the broader Global Burden of Disease (GBD) study 2019, which covered 201 countries from 1990 to 2019. National prevalence, incidence, and DALYs were shown by using ggplot2 and maps packages. Five-year intervals were used for age groupings. The Commonwealth and the World Bank income classifications were used for income grouping.

**Results:**

Globally, there were ~364.58 million prevalent cases (females: 225.16 million), 29.51 million incident cases (females: 17.53 million), and 11.53 million DALYs (females: 7.09 million) due to KOA in 2019. The Western Pacific Region had a high endemicity of ~7,319.87 cases per 100,000 people (7.64%). Japan's prevalence rate (12,610.12 cases per 100,000 population) was 10 times that of Somalia (1,178.23) in 2019. In 200 countries (except the United Arab Emirates), the prevalence, incidence, and DALY rates of KOA in 2019 were higher among females than among males and increased with age up to the oldest age group. The prevalence was highest in the high-middle SDI countries, and the incidence and DALYs were highest in the middle SDI countries.

**Conclusion:**

There was a large burden of KOA worldwide, with some notable intercountry variation. Some countries had 10 times the burden of other countries. Increasing population awareness regarding the prevalence, incidence, and DALYs of KOA with a focus on the population in the Western Pacific Region is needed, particularly for older females. informs health policy development, and contributes to improving the efficiency, equity, and effectiveness of healthcare systems.

## Introduction

Osteoarthritis, a major global health problem, is the most prevalent musculoskeletal disorder and affects over 50 million people in the United States, where it is the leading cause of disability (1). Among them, knee osteoarthritis (KOA) has a high prevalence (2), a chronic disease that results in disability, joint pain, stiffness, and decreased function (3). Incidence and prevalence have often been used to describe the burden of a disease (4). Age, sex, region and economic level are often associated with the incidence and prevalence of a disease. The incidence and prevalence of KOA increases with increasing age (5). After reviewing the literature, we found that the prevalence and incidence of KOA in all age groups were not reported. It is well-known that females suffer more joint and bone problems than males, such as KOA and osteoporosis (6). However, it remains unclear whether there is a true sex difference or whether there are regional or income differences. Thus, clarifying and updating information on the prevalence and incidence of KOA depending on sex, age, income, and region is necessary.

The Global Health Data Exchange (GHDx) is the world's most comprehensive catalog of surveys, censuses, vital statistics, and other health-related data. The GHDx makes publicly available the data for each source included in the Global Burden of Disease (GBD) study (7). The GBD study, the only source of comprehensive quantification of the disabling outcomes of diseases and injuries (8), provides a comprehensive picture of mortality and disability across countries, time periods, ages, and sexes (https://www.healthdata.org/gbd). GBD research incorporates the prevalence, incidence, and disability-adjusted life years (DALYs) of a given disease. By analyzing the GBD database, Safiri et al. (9) reported the global, regional and national burden of osteoarthritis. Zhakhina et al. (10) reported the disease burden of KOA in Kazakhstan. Similarly, Hoveidaei et al. (11) reported the disease burden of KOA in the Middle East, and Song et al. (12) reported the disease burden of KOA in China. However, there is no report on the global and regional burden of KOA, especially no analysis at different levels such as sex, age and economics.

In this study, we screened and analyzed the latest KOA data from the GBD study obtained from the GHDx website, and aimed to determine the global, regional and national prevalence, incidence and DALYs of KOA.

## Methods

### Data sources

Data were downloaded and analyzed using estimates from the GBD study 2019, which covered 201 countries and regions from 1990 to 2019 from the GHDx query tool found at https://vizhub.healthdata.org/gbd-compare/and http://ghdx.healthdata.org/gbd-results-tool (13). Using the GHDx, we identified and compiled a total of 201 countries from 1990 to 2019 regarding KOA prevalence, incidence, and DALY information. DALYs (per 100,000 people) are the sum of years of life lost (YLL) and years lived with disability (YLD) for each location, age group, sex, and year (14). YLL are due to premature mortality. YLL are the multiplication of deaths and a standard life expectancy at death. The standard life expectancy is derived from a life table that contains the lowest observed mortality rate at each age that has been observed in any population >5 million. YLD represents years lived with any short-term or long-term health loss weighted for severity by disability weights (15).

### Data collection

The prevalence, incidence, and DALYs were selected as measurement items, the percentage and rate (per 100,000 population) were selected as metrics, and KOA was selected as the cause. According to various studies, 201 countries were selected as the study locations, and sex indicated males and females. We grouped prevalence, incidence, and DALYs by age group (5-year intervals; < 20, 20–24, 25–29, 30–34, 35–39, 40–44, 45–49, 50–54, 55–59, 60–64, 65–69, 70–74, 75–79, 80–84, 85–89, 90–94, >95 years). An age interval < 30 years was combined in the correlation between age and KOA. The sociodemographic index (SDI; a composite of sociodemographic factors) and income (Commonwealth and World Bank income levels) were selected as variables to evaluate the trend with the prevalence, incidence, and DALYs. The Commonwealth, comprising 54 sovereign nations, in the geographic databases, to categorize and distinguish data pertaining to its member countries.

### Statistical analysis and data presentation

R software (version 4.1.3) was used for data presentation by using ggplot2 and maps packages (available at https://cran.r-project.org/). Prism software (version 9.1.1) was used for the statistical analyses. Values were compared by using Student's *t*-tests for two-group comparisons. A *P-*value < 0.01 was considered significant.

## Results

### Global and regional levels

The global prevalence of KOA was 4,711.84 per 100,000 (4.90%) people for both sexes, 5,838.45 per 100,000 (6.00%) females, and 3,592.35 per 100,000 (3.78%) males. The global incidence of KOA was 381.4 per 100,000 (0.07%) people for both sexes, 454.46 per 100,000 (0.09%) females, and 308.8 per 100,000 (0.06%) males. Global DALYs of KOA were 149.07 per 100,000 (0.45%) people for both sexes, 183.79 per 100,000 (0.59%) females, and 114.56 per 100,000 (0.33%) males. The prevalence varied across geographic areas: the Western Pacific Region had high endemicity, 7,319.87 per 100,000 (7.64%) people for both sexes, 9,543.14 per 100,000 (9.83%) females, and 5,157.03 per 100,000 (5.45%) males, and the African Region had lower endemicity, 1,763.73 per 100,000 (1.82%) people for both sexes, 1,949.20 per 100,000 (1.99%) females, and 1,572.71 per 100,000 (1.63%) males. Interestingly, the regions with the high/low incidence and DALYs were consistent with those regarding prevalence (Table 1).

**Table 1:** Prevalent, incidence, and DALYs for KOA in 2019 for sex, percent and rate by global and WHO regions.

		**Prevalence**			**Incidence**			**DALYs**		
		**Both**	**Females**	**Males**	**Both**	**Females**	**Males**	**Both**	**Females**	**Males**
Global	Percent	4.90%	6.00%	3.78%	0.07%	0.09%	0.06%	0.45%	0.59%	0.33%
	Rate	4,711.84	5,838.45	3,592.35	381.40	454.46	308.80	149.07	183.79	114.56
African Region	Percent	1.82%	1.99%	1.63%	0.03%	0.03%	0.03%	0.12%	0.14%	0.10%
	Rate	1,763.73	1,949.20	1,572.71	174.75	190.32	158.72	56.17	61.78	50.39
Western Pacific Region	Percent	7.64%	9.83%	5.45%	0.13%	0.16%	0.10%	0.86%	1.23%	0.56%
	Rate	7,319.87	9,543.14	5,157.03	558.28	701.27	419.18	234.01	303.42	166.48
Eastern Mediterranean Region	Percent	2.40%	2.61%	0.25%	0.04%	0.04%	0.04%	0.22%	0.25%	0.19%
	Rat	2,284.79	2,522.26	79.58	227.09	247.52	208.20	72.59	79.58	66.13
South-East Asia Region	Percent	3.49%	4.23%	2.77%	0.06%	0.07%	0.05%	0.33%	0.41%	0.25%
	Rate	3,399.97	4,156.21	2,665.90	302.33	359.69	246.64	106.71	129.71	84.38
Region of the Americas	Percent	5.96%	7.02%	4.83%	0.08%	0.09%	0.07%	0.59%	0.75%	0.44%
	Rate	5,671.63	6,785.96	4,515.40	446.58	515.64	374.93	177.09	211.04	141.86
European Region	Percent	6.76%	8.09%	5.30%	0.09%	0.11%	0.08%	0.62%	0.80%	0.45%
	Rate	6,424.67	7,830.18	4,942.06	476.46	549.40	399.51	201.91	244.96	156.50

DALYs, disability-adjusted life years; Rate (per 100,000); Both: females & males.

### National level

**Figure 1:**
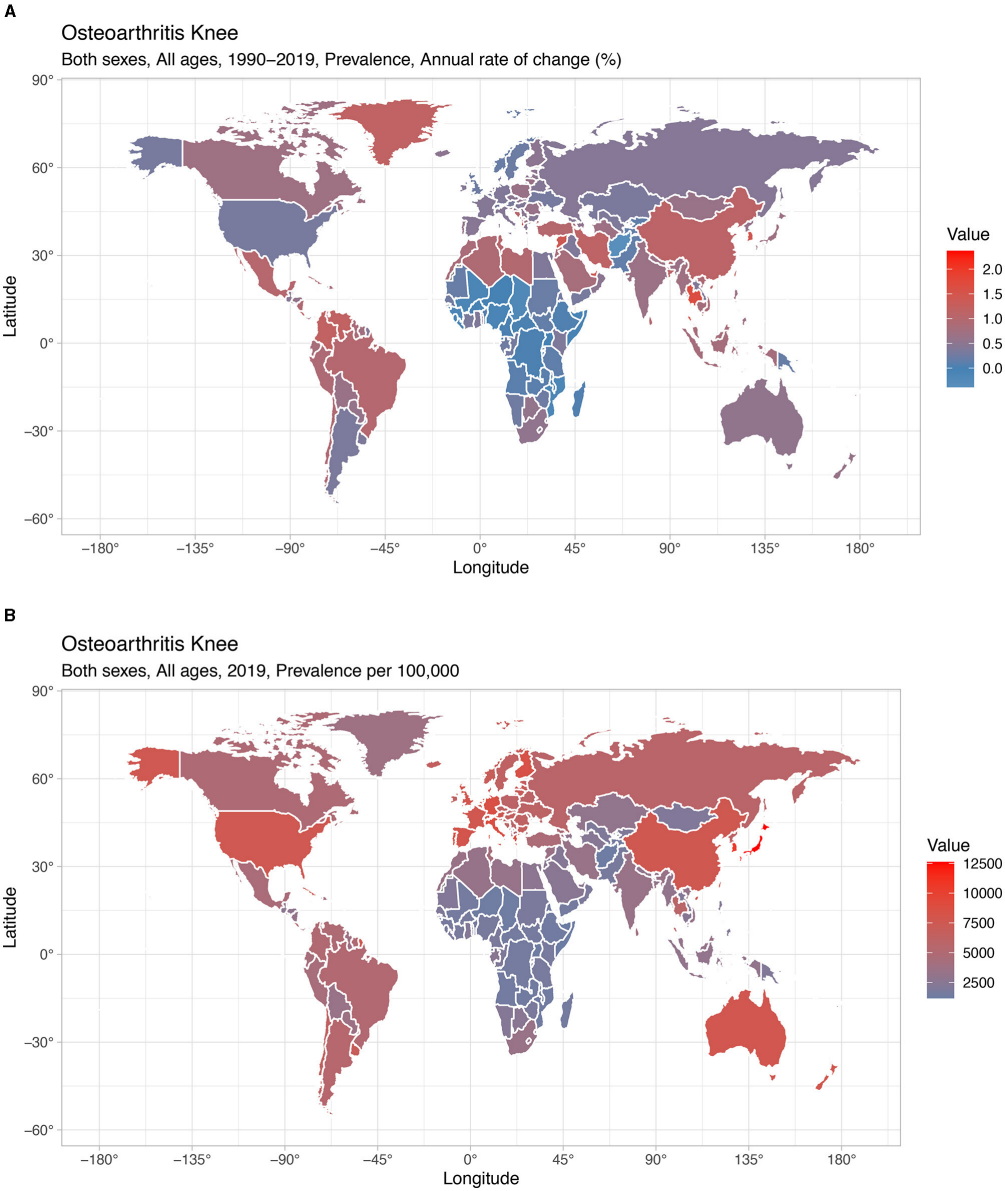
National prevalence of KOA. **(A)** Prevalence from 1990 to 2019. **(B)** Prevalence in 2019.

**Figure 2:**
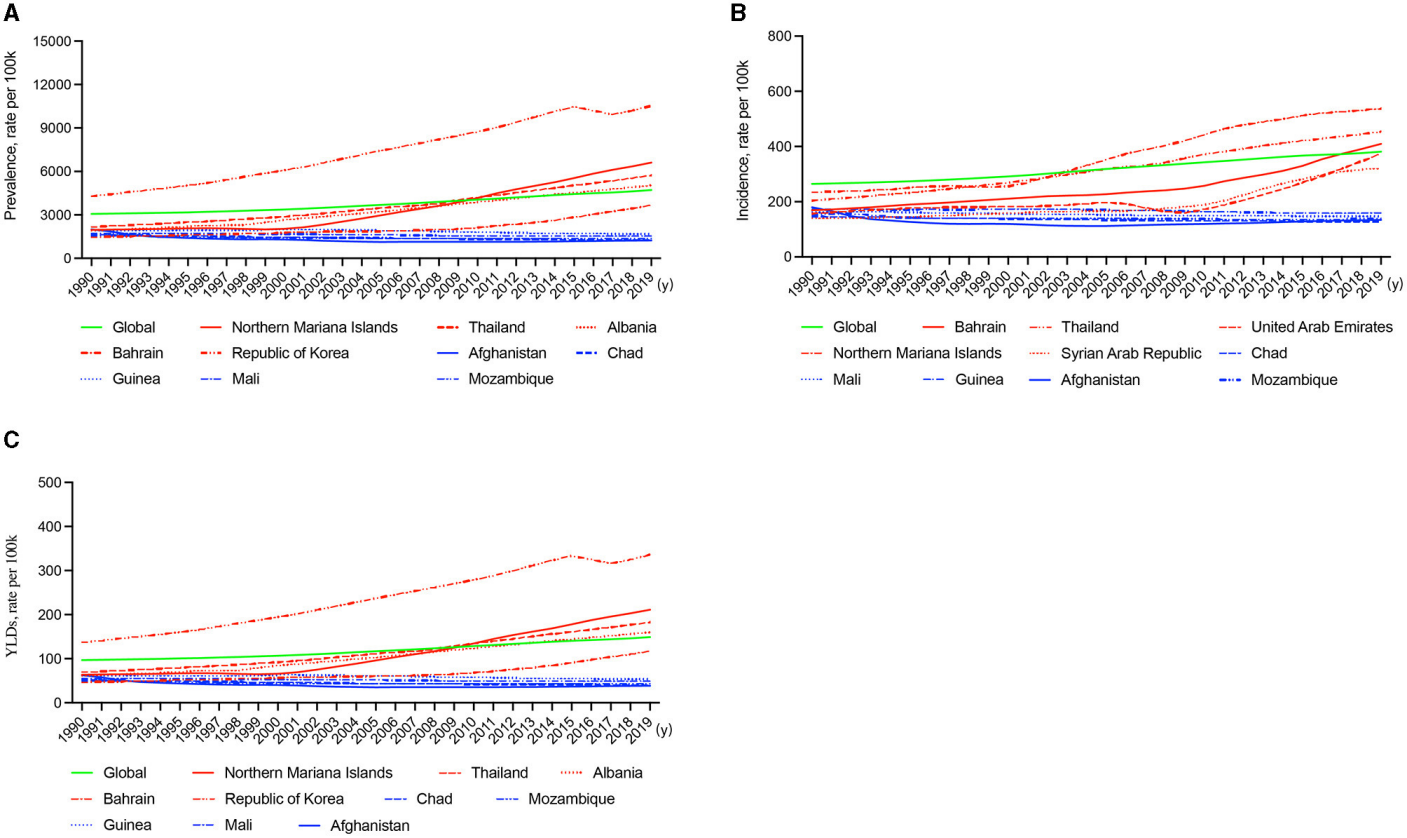
Line graphs of the global prevalence, incidence, and DALYs (green line) and the five countries with the highest (red line) and lowest rankings (blue line) of KOA, 1990–2019. **(A)** Prevalence. **(B)** Incidence. **(C)** DALYs.

National prevalence rate estimates of KOA ranged from 1,178.23 (Somalia) to 12,610.12 (Japan) cases per 100,000 population in 2019 and from −0.38 (Afghanistan) to 2.37 (Northern Mariana Islands) cases per 100,000 population in 1990–2019 (Figure 1). The five countries with the greatest KOA prevalence rates in 1990–2019 were the Northern Mariana Islands (2.37), Thailand (1.65), Albania (1.61), Bahrain (1.49), and the Republic of Korea (1.45), and the five countries with the lowest KOA prevalence rates in 1990–2019 were Afghanistan (−0.38), Chad (−0.20), Mozambique (−0.14), Guinea (−0.13), and Mali (−0.11) (Figure 2A).

The national incidence rate of KOA ranged from 125.99 (Somalia) to 777.73 (Republic of Korea) cases per 100,000 population in 2019 and from −0.26 (Afghanistan) to 1.42 (Bahrain) cases per 100,000 population in 1990–2019 (Supplementary Figure 1). The five countries with the highest KOA incidence rates in 1990–2019 were Bahrain (1.42), United Arab Emirates (1.39), the Syrian Arab Republic (1.30), Northern Mariana Islands (1.29), and Thailand (1.23), and the five countries with the lowest KOA incidence rates in 1990–2019 were Afghanistan (−0.26), Chad (−0.12), Mozambique (−0.12), Mali (−0.09), and Guinea (−0.08) (Figure 2B).

The national DALY rate of KOA ranged from 37.59 (Somalia) to 397.68 (Japan) cases per 100,000 population in 2019 and from −0.38 (Afghanistan) to 2.32 (Northern Mariana Islands) in 1990–2019 (Supplementary Figure 2). The five countries with the highest KOA DALY rates in 1990–2019 were the Northern Mariana Islands (2.32), Thailand (1.65), Albania (1.60), Bahrain (1.48), and the Republic of Korea (1.45), and the five countries with the lowest KOA DALY rates in 1990–2019 were Afghanistan (−0.38), Chad (−0.20), Mozambique (−0.14), Guinea (−0.13), and Mali (−0.11) (Figure 2C).

### Sex- and age-based patterns

**Figure 3:**
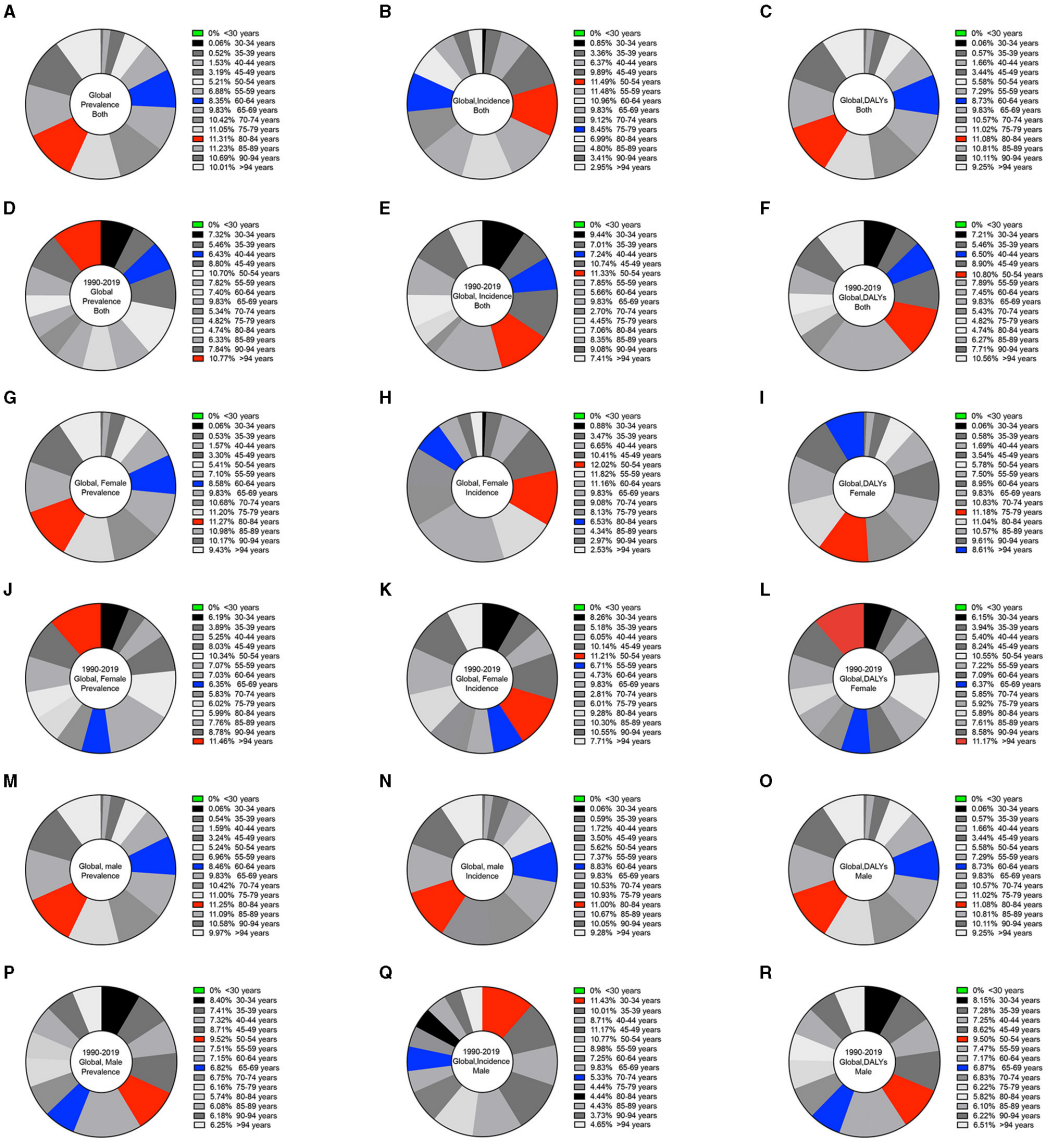
Pie charts of the global prevalence, incidence, and DALYs by sex and different age bin ranges for KOA. **(A)** Global prevalence for both sexes in 2019. **(B)** Global incidence for both sexes in 2019. **(C)** Global DALYs for both sexes in 2019. **(D)** Global prevalence for both sexes from 1990 to 2019. **(E)** Global incidence for both sexes from 1990 to 2019. **(F)** Global DALYs for both sexes from 1990 to 2019. **(G)** Global prevalence among females in 2019. **(H)** Global incidence among females in 2019. **(I)** Global DALYs among females in 2019. **(J)** Global prevalence among females from 1990 to 2019. **(K)** Global incidence among females from 1990 to 2019. **(L)** Global DALYs among females from 1990 to 2019. **(M)** Global prevalence among males in 2019. **(N)** Global incidence among males in 2019. **(O)** Global DALYs among males in 2019. **(P)** Global prevalence among males from 1990 to 2019. **(Q)** Global incidence among males from 1990 to 2019. **(R)** Global DALYs among males from 1990 to 2019.

Regarding sex, the global prevalence of KOA in 2019 was higher among females (5,838.45 per 100,000) than among males (3,592.35 per 100,000), with the highest ratio in the 80–84 age group (23,532.52 per 100,000) and the median ratio in the 60–64 age group (17,382.75 per 100,000) (Figure 3A). The global incidence of KOA in 2019 was higher among females (454.46 per 100,000) than among males (308.80 per 100,000), with the highest ratio in the 50–54 age group (1,166.95 per 100,000) and the median ratio in the 75–79 age group (857.51 per 100,000) (Figure 3B). The global DALYs were higher among females (183.79 per 100,000) than among males (114.56 per 100,000), with the highest ratio in the 80–84 age group (702.32 per 100,000) and the median ratio in the 60–64 age group (553.32 per 100,000) (Figure 3C). Regarding the global prevalence of KOA in 1990–2019, the highest ratio was in the >94 age group (10.77% in all groups), and the median ratio was in the 40–44 age group (6.43%) (Figure 3D). The highest ratio was in the 50–54 age group (11.33%), and the median ratio was in the 40–44 age group (7.24%) for global incidence (Figure 3E). The highest ratio was in the 50–54 age group (10.08%), and the median ratio was in the 40–44 age group (6.50%) for global DALYs (Figure 3F).

Among females, the global prevalence of KOA in 2019 was the highest in the 80–84 age group (23,532.52 per 100,000) and the median in the 60–64 age group (17,382.75 per 100,000) (Figure 3G). The highest ratio was in the 50–54 age group (1,384.37 per 100,000), and the median ratio was in the 80–84 age group (752.18 per 100,000) for global incidence (Figure 3H). The highest ratio was in the 75–79 age group (815.11 per 100,000), and the median ratio was in the >94 age group (628.23 per 100,000) for global DALYs (Figure 3I). Regarding the global prevalence of KOA in 1990–2019, the highest ratio was in the >94 age group (11.46%), and the median ratio was in the 65–69 age group (6.35%) (Figure 3J). The highest ratio was in the 50–54 age group (11.21%), and the median ratio was in the 55–59 age group (6.71%) for global incidence (Figure 3K). The highest ratio was in the >94 age group (11.17%), and the median ratio was in the 65–69 age group (6.37%) for global DALYs (Figure 3L).

Among males, the global prevalence of KOA in 2019 was the highest in the 80–84 age group (18,647.49 per 100,000) and the median in the 60–64 age group (14,014.29 per 100,000) (Figure 3M). The highest ratio was in the 80–84 age group (650.55 per 100,000), and the median ratio was in the 60–64 age group (931.34 per 100,000) for global incidence (Figure 3N). The highest ratio was in the 80–84 age group (558.76 per 100,000), and the median ratio was in the 60–64 age group (448.47 per 100,000) for global DALYs (Figure 3O). The global prevalence of KOA in 1990–2019 was highest in the 50–54 age group (9.52%) and median in the 65–69 age group (6.82%) (Figure 3P). The highest ratio was in the 30–34 age group (11.43%), and the median ratio was in the 70–74 age group (5.33%) for global incidence (Figure 3Q). The highest ratio was in the 50–54 age group (9.50%), and the median ratio was in the 65–69 age group (6.87%) for global DALYs (Figure 3R).

**Figure 4:**
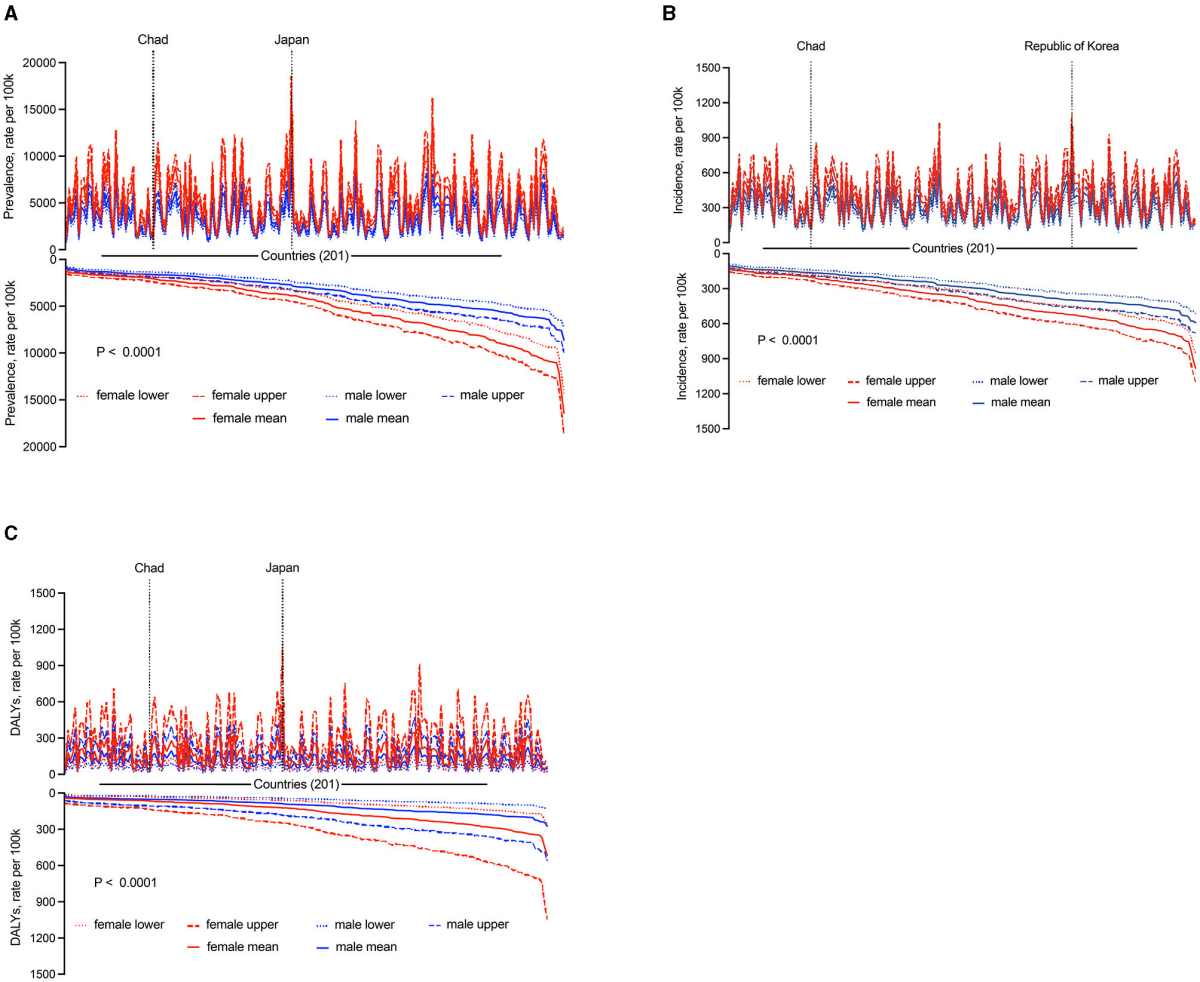
National sex differences in KOA in 2019. **(A)** Prevalence. **(B)** Incidence. **(C)** DALYs.

The prevalence, incidence, and DALYs were higher among females than among males in the 200 countries (*P* < 0.0001) (Figure 4). However, in the United Arab Emirates, these indicators were higher among males than among females (Supplementary Table 1).

### SDI- and income-based patterns

**Figure 5:**
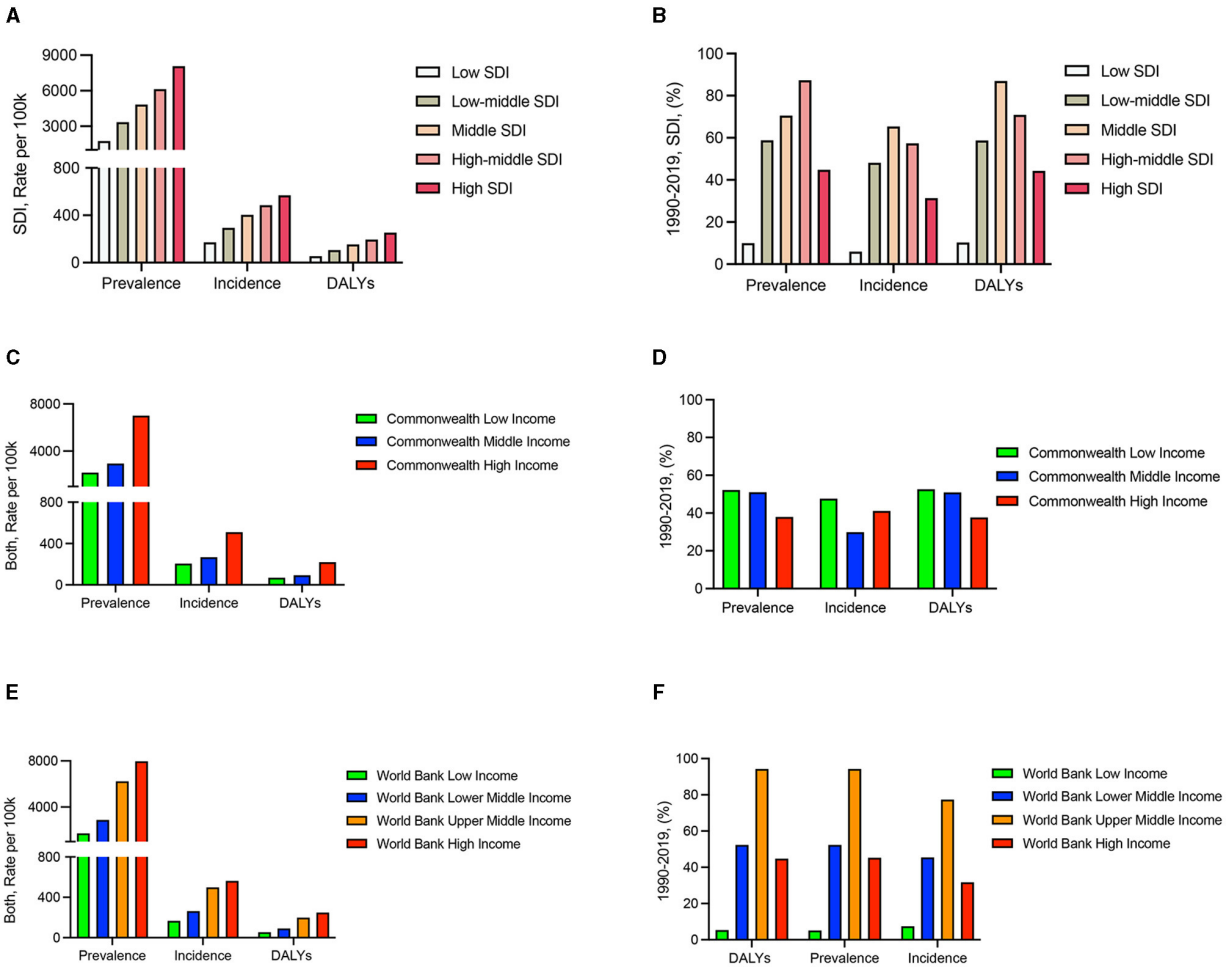
Prevalence, incidence, and DALYs of SDI and income among patients with KOA. **(A)** SDI in 2019. **(B)** SDI from 1990 to 2019. **(C)** Commonwealth income classifications in 2019. **(D)** Commonwealth income classifications from 1990 to 2019. **(E)** World Bank income classifications in 2019. **(F)** World Bank income classifications from 1990 to 2019.

In order to estimate the association between the SDI and the ratio of the prevalence, incidence, and DALYs. The SDI had a positive association with the prevalence rate in 2019 and no association with sex (Figure 5A; Supplementary Figures 3A, 4A). In 1990–2019, the prevalence was highest in the high-middle SDI group, and the incidence and DALYs were highest in the middle SDI group for both sexes (Figure 5B); the prevalence, incidence, and DALYs were highest in the middle SDI group for females (Supplementary Figure 3B); notably, negative values were obtained for the incidence among males (−1.08%) (Supplementary Figure 4B).

Then, we assessed the strength of the association of income with KOA prevalence, incidence, and DALYs through Commonwealth and World Bank income classifications. Our results demonstrated that income was positively related to the prevalence, incidence, and DALYs in 2019 (Figures 5C, E) and independent of sex (Supplementary Figures 3C, E, 4C, E). In 1990–2019, slightly increased rates were seen in Commonwealth regions (Figure 5D) and regions classified as having high incomes according to the World Bank (Figure 5F); this finding was even more pronounced among females (Supplementary Figures 3D, F, 4D, F).

## Discussion

This study provided information on the KOA prevalence, incidence, and DALYs between 1990 and 2019 at global, regional and national levels according to age, sex, SDI, and income as defined by the GBD study. In 2019, KOA accounted for 364.58 million prevalent cases (females: 225.16 million), 29.51 million incident cases (females: 17.53 million), and 11.53 million DALYs (females: 7.09 million). The prevalence varied across geographic areas: the Western Pacific Region had high endemicity. In the United Arab Emirates, these indicators were higher among males than among females. These data highlight the significant but underrecognized global burden of KOA.

Osteoarthritis is a common degenerative joint disease, and the knee is the most common joint affected (16). Quantifying both incidence rates and burden is important (17). However, there is no good way to predict the prevalence and incidence of KOA.

The prediction of disease prevalence and incidence based on GBD studies has been proven to be effective in many studies (18–21). On a global level, the results of our study showed that the estimated prevalence of KOA was ~4.90%, which is higher than that reported in the literature, ~3.80% (22). This finding suggests that the annual number of KOA cases has increased. This is consistent with our findings that the prevalence of KOA increased by 54.17% per year, the incidence increased by 49.14% per year, and the DALYs increased by 126.93% per year from 1990 to 2019. At the regional level, the Western Pacific Region had high endemicity, with 7,319.87 cases per 100,000 people (7.64%). This study updates the previous view that there are no regional differences in the occurrence of KOA (23). In the Western Pacific Region, from 1990 to 2019, the country with the highest prevalence, incidence and DALYs of KOA was Thailand. In 2019, the country with the highest prevalence was Japan (~12,610.11 cases per 100,000 people), the country with the highest incidence was the Republic of Korea (~777.73 cases per 100,000 people), and the country with the highest DALYs was Japan (~397.68 DALYs per 100,000 people). Established clinical risk factors for KOA include older age, female sex, and smoking status (24). White individuals have much lower smoking rates than Japanese and Korean individuals (25). This may partially explain the situation of KOA in Japan and the Republic of Korea.

With rapid population aging, the incidence of KOA increased yearly (26). To further clarify the correlation between age and KOA, populations were grouped by 5-year age categories to allow for the effects of age. The results showed that no KOA cases occurred among individuals younger than 30 years, which is probably the first time that this has been reported. The prevalence of KOA among people over 60 years of age accounted for 82.89% of the total prevalence, with more than 37% of those in this age group showing radiographic disease (27). The prevalence of KOA was highest among 80–84-year-olds with no sex difference, the incidence was highest among 50–54-year-olds with no sex difference, and the DALYs were highest among 80–84-year-olds, with females having a greater number of DALYs than males in 2019. Multiple studies have reported an increase in the prevalence of KOA among females compared to males (6, 28, 29). Overall, our findings were consistent with the findings of the prior study. However, it is worth noting that the increase in the prevalence, incidence, and DALY rates of KOA were greater among men than among women in the United Arab Emirates.

Previous studies have shown that the SDI is associated with the prevalence and incidence of diseases (30, 31), but this remains unknown for KOA. In this study, first, the prevalence, incidence and DALYs were positively correlated with the SDI in 2019. The crude adult prevalence increased more than the age-standardized prevalence in regions that had substantial aging—e.g., in high-income regions (32). At least some of the differences in the incidence may be due to a lack of diagnostic abilities in lower-SDI countries compared with high-SDI countries and do not necessarily reflect differences in disease biology (8, 30, 31). Second, middle-SDI countries had a higher prevalence and the highest incidence and DALY rates from 1990 to 2019. A similar pattern emerged with the SDI as the measure of economic development (33), and this increase can be explained by changes in diagnostic criteria and increased public awareness of the disease. The burden of KOA is daunting in light of substantial economic stresses (34). Studies have shown that trends in incidence vary between income groups (35). However, the prevalence, incidence, and DALY rates of KOA among countries with different incomes are unknown. Our results indicated that a high national income is closely tied to a high prevalence, incidence, and DALY rates of KOA regardless of the Commonwealth or World Bank income classifications.

With a rapidly aging global population, the demand for health services will require policy-makers to predict changes in diseases (7). In areas where specialists are difficult to reach, the unknown incidence and prevalence of KOA may lead to a lower level of attention in these areas, which may lead to a more serious development of the disease. Considering the effectiveness of current treatment, the major challenge faced by KOA research is early diagnosis and identification of patients with fast progression of KOA (36). Our results described the prevalence, incidence, and DALY rates of KOA for different sexes, ages, regions, and income groups. Our present study has important information for future KOA studies.

Our research has some advantages and limitations. This study reports the levels and trends of the KOA burden in 201 countries and regions from 1990 to 2019. We provide researchers with data on the prevalence of KOA worldwide, as well as emerging cases, identifying a high prevalence in the Western Pacific region. In the United Arab Emirates, the prevalence, rates, and DALY rates of KOA were higher among males than among females. The limitations of this study are consistent with those previously reported (37). First, all data may be affected by test deviations, and some countries have difficulties with data quality, particularly low- and middle-income countries. Second, because the data source relies on patients with medical records, the reported estimates may underestimate the true incidence. Third, data deficiencies may occur, which can only be mitigated by improved epidemiological evidence.

## Conclusion

This study found that the burden of KOA varied considerably between countries, sexes, ages and income levels. Notably, the prevalence, incidence, and DALY rates of KOA increased yearly from 1990 to 2019, females were significantly higher than males in most areas (except the United Arab Emirates). Clear differences in the prevalence, incidence, and DALY rates of KOA in different regions and different populations will help to increase the attention of the population and national policy-makers regarding KOA and reduce the future burden of the disease.

## Data availability statement

The original contributions presented in the study are included in the article/Supplementary material, further inquiries can be directed to the corresponding author.

## Ethics statement

The GBD study's protocol has been approved by the Research Ethics Board at the University of Washington.

## Author contributions

EL: Formal analysis, Investigation, Methodology, Software, Visualization, Writing – original draft. JT: Data curation, Formal analysis, Methodology, Validation, Writing – original draft. KX: Supervision, Validation, Writing – review & editing. YP: Data curation, Formal analysis, Investigation, Writing – original draft. PX: Conceptualization, Funding acquisition, Project administration, Supervision, Validation, Writing – review & editing.
