# Supplementary figures and images for: Global burden and socioeconomic impact of knee osteoarthritis: a comprehensive analysis
Source: Front Med (Lausanne). 2024 May 16;11:1323091. doi: 10.3389/fmed.2024.1323091 (PMC11137242; doi:10.3389/fmed.2024.1323091)

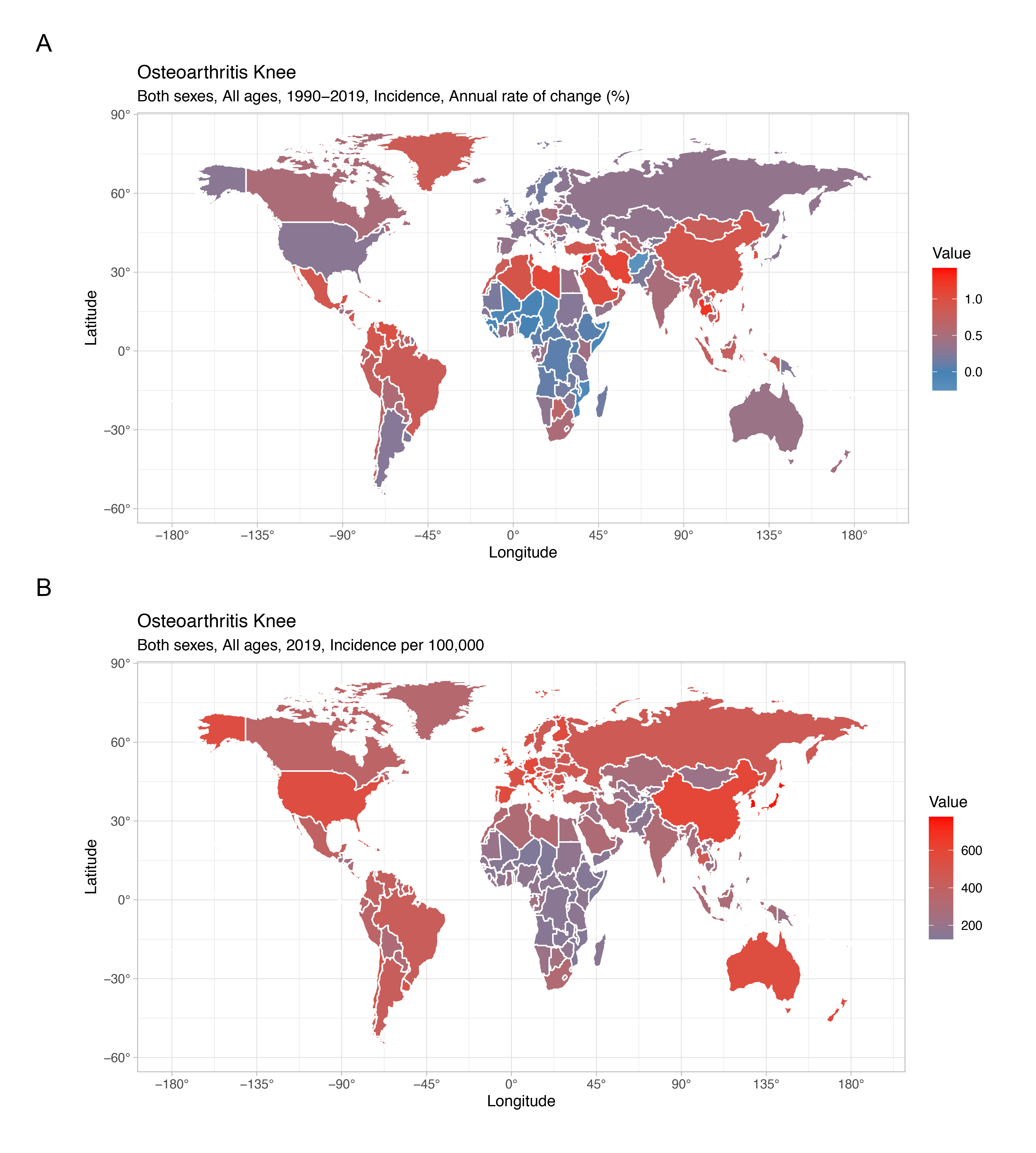

Supplement: Supplementary file 1 [file Image_1.TIF]

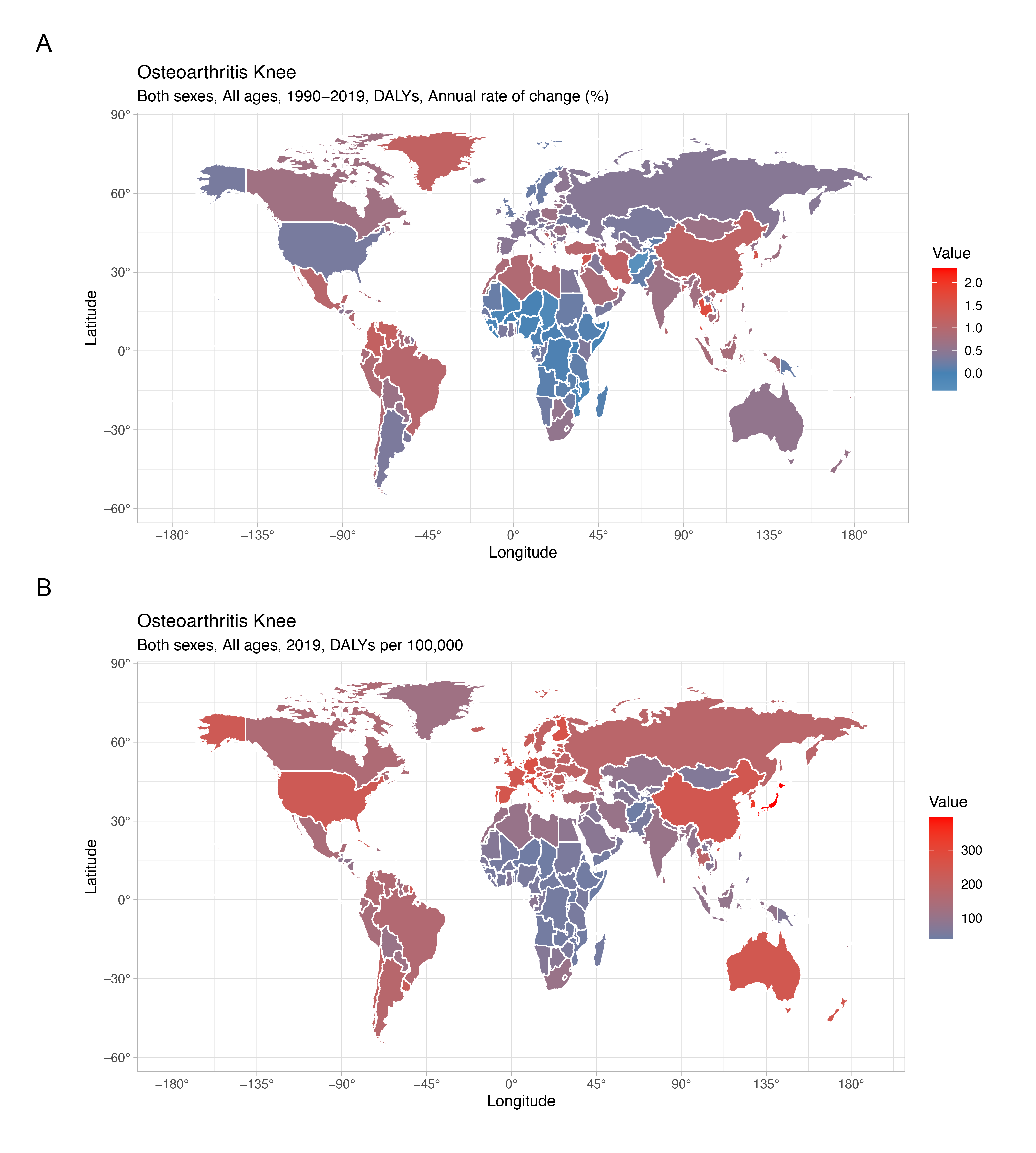

Supplement: Supplementary file 2 [file Image_2.TIF]

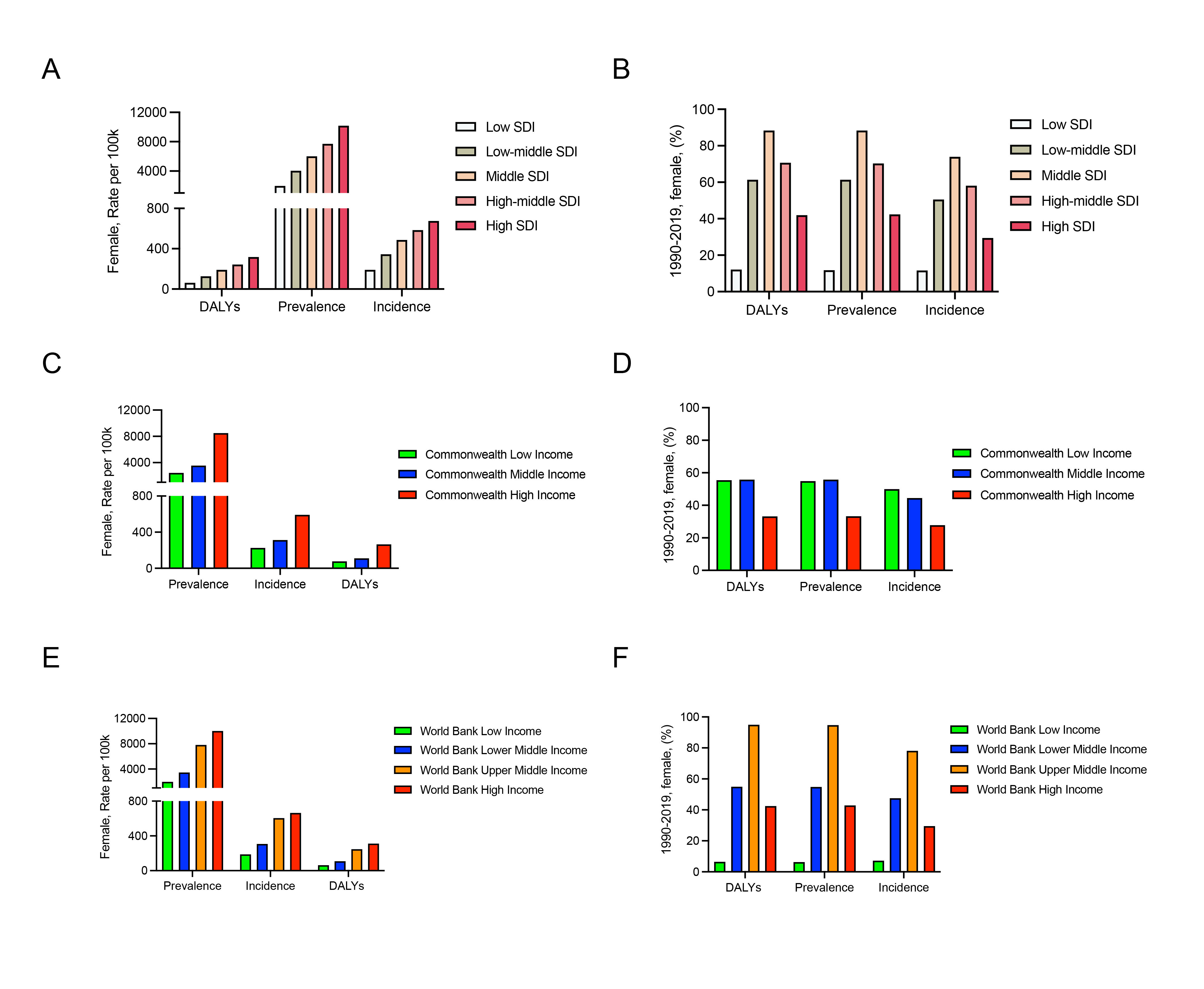

Supplement: Supplementary file 3 [file Image_3.TIF]

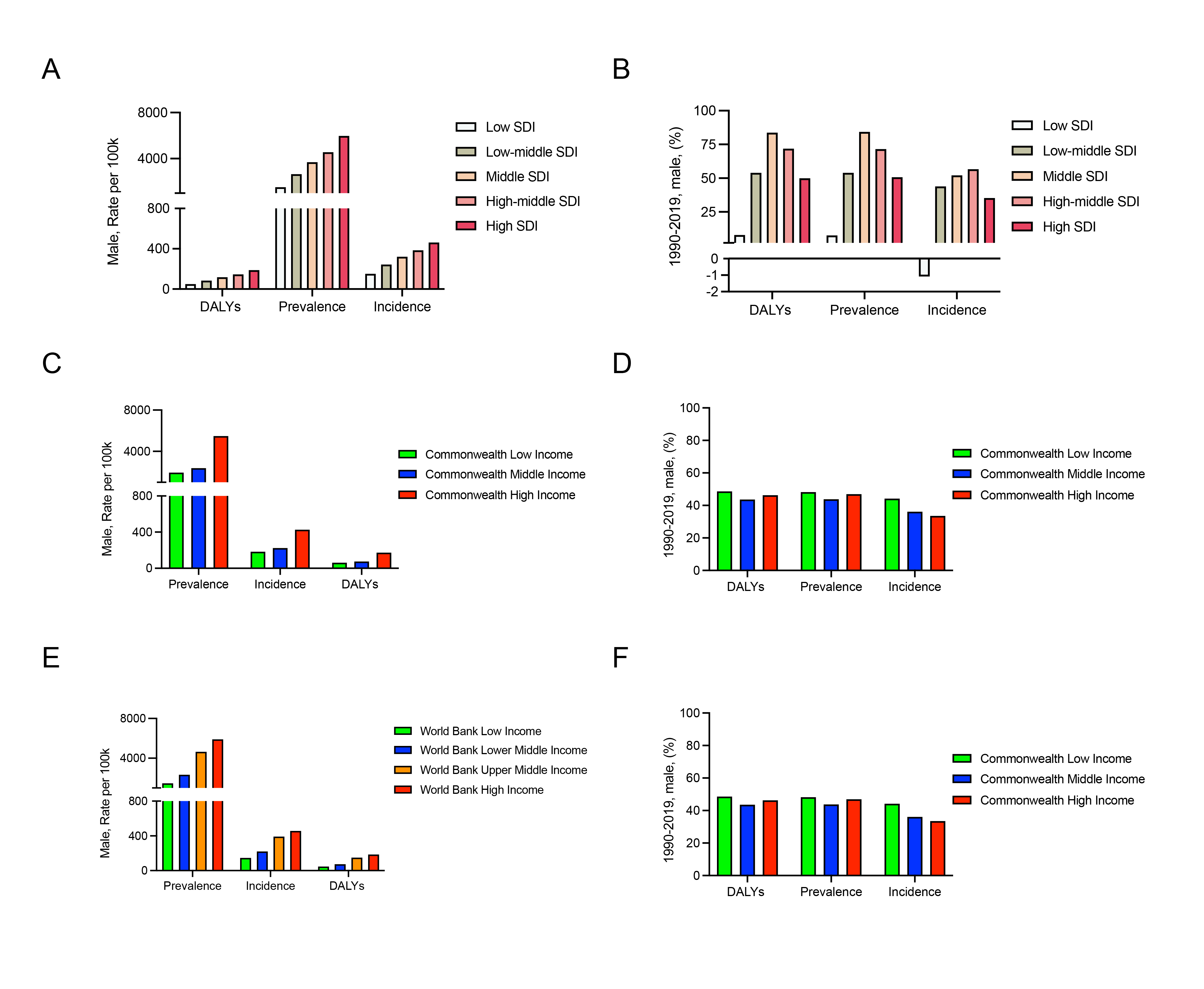

Supplement: Supplementary file 4 [file Image_4.TIF]
