# Supplementary material for: Global burden and socioeconomic impact of knee osteoarthritis: a comprehensive analysis
Source: Front Med (Lausanne). 2024 May 16;11:1323091. doi: 10.3389/fmed.2024.1323091 (PMC11137242; doi:10.3389/fmed.2024.1323091)
Supplement: Supplementary file 5 [file Table_1.DOCX]

Supplementary material

Figures

Supplemental Figure 1. National incidence of KOA. A, Incidence from 1990 to 2019. B, Incidence in 2019

Supplemental Figure 2. National DALYs of KOA. A, DALYs from 1990 to 2019. B, DALYs in 2019

Supplemental Figure 3. Prevalence, incidence, and DALYs of SDI and income among female patients with KOA. A, SDI in 2019. B, SDI from 1990 to 2019. C, Commonwealth income classifications in 2019. D, Commonwealth income classifications from 1990 to 2019. E, World Bank income classifications in 2019. F, World Bank income classifications from 1990 to 2019.

Supplemental Figure 4. Prevalence, incidence, and DALYs of SDI and income among male patients with KOA. A, SDI in 2019. B, SDI from 1990 to 2019. C, Commonwealth income classifications in 2019. D, Commonwealth income classifications from 1990 to 2019. E, World Bank income classifications in 2019. F, World Bank income classifications from 1990 to 2019.
